# Virtual Reality as Exposure Therapy in the Treatment of Blood-Injection-Injury Phobias

**DOI:** 10.1192/j.eurpsy.2024.1472

**Published:** 2024-08-27

**Authors:** N. Halouani, N. Boussaid, M. Turki, M. Barkallah, S. Ellouze, J. Aloulou

**Affiliations:** psychiatrie B, Hedi Chaker university hospital, Sfax, Tunisia

## Abstract

**Introduction:**

Virtual Reality (VR) is a transformative technology that facilitates the development of immersive virtual environments. Its application is steadily growing within Cognitive Behavioral Therapy (CBT) techniques, notably in virtuo exposure therapy. This is particularly evident in the treatment of specific phobias, with a specific focus on addressing blood-injection-injury phobias.

**Objectives:**

The objective of our study is to design a treatment protocol for patients suffering from blood phobia based on VR.

**Methods:**

We used the following scales:

- Fear Survey Schedule-III (FSS-III) and the Injection Phobia Scale (IPS) for psychometric evaluation of the intensity of avoidance fear.
- Questionnaire on cybersickness: to identify potential adverse effects of exposure to virtual reality.

To conduct a functional analysis of phobias, we used the SECCA grid and the SORC grid.

**Results:**

The therapeutic protocol stages of VR for a patient suffering from Blood-Injection-Injury Phobia (BIIP) are as follows:

1. Collection of sociodemographic and clinical data.
2. Functional analysis to identify triggering factors, contributing factors, and consequences of behavior. The SECCA or SORC grid can help in conducting this functional analysis.
3. Psychometric evaluation of the intensity of avoidance fear using the three scales: FSS-III, IPS, and the cybersickness scale.
4. Patient education on the mechanisms of the phobia.
5. Setting of objectives.
6. Therapeutic contract.
7. The Protocol :
8. Cognitive approach: identification of automatic thoughts and replacement with more rational thoughts.
9. Behavioral approach: Progressive exposure, controlled immersion of the patient in virtual environments corresponding to situations that trigger their phobia. This exposure is coupled with relaxation.

The treatment continues with regular follow-up to ensure the consolidation of progress and to adjust strategies.

For relapse prevention, simple measures ,like personalized exercises to be done by the patient, can favor the long-term maintenance of the acquired skills.

**Conclusions:**

Virtual reality exposure therapies (in virtuo) are as effective as in-vivo therapies. Besides, they offer a significant advantage over the latter as they facilitate access to stimuli or anxiety-provoking situations that are difficult to access or control in the real world.

**Disclosure of Interest:**

None Declared

